# Delayed villous maturation with and without fetal demise: A case report of three successive pregnancies

**DOI:** 10.1111/jog.16308

**Published:** 2025-05-06

**Authors:** M. C. Marijnen, W. Ganzevoort, S. J. Gordijn, L. E. van der Meeren

**Affiliations:** ^1^ Department of Obstetrics and Gynecology, Amsterdam University Medical Centers University of Amsterdam Amsterdam The Netherlands; ^2^ Amsterdam Reproduction & Development Research Institute Amsterdam The Netherlands; ^3^ Department of Obstetrics and Gynecology University Medical Center Groningen, University of Groningen Groningen The Netherlands; ^4^ Department of Pathology Leiden University Medical Center Leiden Leiden The Netherlands; ^5^ Department of Pathology Erasmus Medical Center, University Medical Center Rotterdam The Netherlands

**Keywords:** maturation disorders, placental pathology, stillbirth

## Abstract

This case study examined three subsequent pregnancies with delayed villous maturation (DVM) resulting in infants either large for gestational age (LGA) or appropriate for gestational age. A perinatal pathologist histopathologically reviewed the placentas using the Amsterdam Consensus Criteria. The first pregnancy ended in a term fetal demise of an LGA infant due to severe asphyxia associated with idiopathic DVM. Due to the history of stillbirth and DVM, labor was induced at 36 weeks of gestation in the second and third pregnancies. The second and third pregnancies resulted in liveborn infants with varying weight profiles despite similar placental lesions. All three placentas showed DVM with positive CD15 immunostaining. Additionally, the second and third placentas exhibited villitis of unknown etiology. This case report underscores the importance of structured histologic placental examination following complicated pregnancies by a perinatal pathologist.

## INTRODUCTION

The placenta plays a vital role in pregnancy, serving as a lifeline between mother and fetus, supporting fetal development and growth. The placenta acts as a barrier, protecting the fetus from harmful substances. The immunological processes are delicate and complex due to the semi‐allogenic fetus, with on the one hand the need for adequate immune responses against microbes and viruses and on the other hand the regulated interaction with maternal immune responses, preventing the mother's immune system from attacking the fetal‐placental unit.^1^ Placental pathology can have significant implications for maternal and fetal health.^2^

Histopathologic placental examination contributes to a better understanding of the pregnancy course, pathophysiology of underlying disease, and pregnancy outcomes.^3^ Particularly, in pregnancies with severe adverse outcomes, such as early onset fetal growth restriction (FGR) or fetal demise prognostic information for maternal and neonatal (long‐term) health may be provided by the placenta.^3^ Placental examination results can guide counseling, monitoring, management, and preventive interventions in subsequent pregnancies to improve outcomes.^3^

Standardized reporting of placental lesions is highly encouraged to ensure reproducibility.^4^, ^5^, ^6^ The Amsterdam Consensus Statement established consistent diagnostic criteria for identifying and reporting clinically significant lesions.^4^ Placental lesions can be separated into four major categories: maternal vascular malperfusion (MVM), fetal vascular malperfusion (FVM), acute inflammation (AI) and chronic inflammation (CI), or other significant pathology.^5^ Previous studies showed the importance of overlapping patterns of placental injury on clinical outcomes.^5^, ^6^, ^7^ Delayed villous maturation (DVM) is a maturation disorder of the placenta and is classified under the category of “other significant pathology” according to the Freedman‐Ernst subclassification.^5^ DVM is typically observed after 36 weeks of gestation and is characterized by a uniform villous population, a reduced number of vasculosyncytial membranes for the corresponding gestational age, a continuous cytotrophoblast layer, and centrally located capillaries.^8^ A study by Jaiman et al. found that placentas from women with fetal death are 44 times more likely to exhibit disorders of villous maturation compared to those from women with normal pregnancy outcomes.^9^

In this report, we describe placental pathology findings of three subsequent pregnancies with a distinctive course and fetal growth pattern, of which the first pregnancy ended in late fetal demise. The placentas were evaluated by a perinatal pathologist. Our aim was to use standardized placental pathology reporting to understand the prenatal findings and outcomes of the three pregnancies of this patient with three placentas available for histologic evaluation.

## CASE REPORT

### Case description

We report three subsequent pregnancies of a Caucasian female. The history started with a fetal demise. During her first ongoing pregnancy, she was a 32‐year‐old gravida 2 para 0. In her obstetric history, she had a curettage after an incomplete miscarriage at 9 weeks of gestation. There was no other relevant medical history. Her body mass index was 20 kg/m^2^. In the first ongoing pregnancy, with a vanishing twin, combined first‐trimester screening was performed and indicated no increased risk of Trisomy 13, 18, or 21. During the second trimester, macrosomia was suspected based on ultrasound scans with an estimated fetal weight (EFW) between the 95th and 100th percentile. Oral glucose tolerance testing with 75 g of glucose was conducted on two separate occasions, and the results from both tests were negative, suggesting the absence of gestational diabetes (GDM). The patient received primary care from a midwife at a midwifery practice. Fetal growth was monitored due to suspected macrosomia, with follow‐up ultrasound scans showing consistent fetal growth between the 95th and 100th percentile. Doppler measurements or routine cardiotocography were not performed. There were no other notable complications throughout the course of pregnancy. Nonetheless, at 38 weeks and 4 days, a fetal demise occurred. The fetal demise was diagnosed by the midwife following an episode of reduced fetal movements. The patient was referred to a secondary care center, where the diagnosis was confirmed by a gynecologist. She reported no blood loss, abdominal pain, or other symptoms. An abdominal ultrasound showed no signs of fetal hydrops, pericardial effusion, or ascites. After induction of labor, she delivered a stillborn LGA male infant with a birthweight of 4065 g (95th percentile). No visible congenital anomalies were observed, and a fetal autopsy was not performed. One year later, she conceived again. The non‐invasive prenatal testing result was normal. Because of her previous stillbirth, low‐dose aspirin prophylaxis was pragmatically initiated from 17 to 35 weeks, following the start of her care at a tertiary care center. Macrosomia was again suspected from 27 weeks, with an EFW at the 99th percentile and fetal abdominal circumference (FAC) above the 100th percentile. Oral glucose tolerance testing results were normal. From 27 weeks onward, weekly assessments of the amniotic fluid index, the umbilical artery pulsatility index, middle cerebral artery pulsatility index, and the cerebroplacental ratio were performed. All measurements remained within normal ranges throughout the pregnancy, indicating no signs of placental insufficiency. Biometric measurements at 31 and 34 weeks followed a consistent growth trajectory, with EFW at the 99th percentile and FAC above the 100th percentile. Routine cardiotocography was not performed, though cardiotocography at 36 weeks was normal. Given her history of stillbirth, shared decision‐making, supported by a multidisciplinary consultation of obstetricians and neonatologists, led to timely induction at 36 weeks and 2 days. She gave birth to a healthy, LGA, male infant with a birthweight of 3960 g (>99th percentile). After this pregnancy, she conceived once more. Low‐dose aspirin prophylaxis was again pragmatically initiated from 19 to 34 weeks. Non‐invasive prenatal testing was normal. No signs of macrosomia were observed, and the fetus exhibited a normal growth trajectory. At 20 weeks of gestation, EFW was at the 52nd percentile and FAC at the 56th percentile. By 28 weeks, EFW increased to the 70th percentile and FAC to the 83rd percentile. At 31 weeks, the EFW was at the 61st percentile and FAC at the 70th percentile. By 34 weeks, EFW was at the 89th percentile and FAC at the 98th percentile. Doppler measurements and the amniotic fluid index at 28, 31, and 35 weeks were within normal ranges. At 36 weeks, her cardiotocography was normal. Considering her obstetric history and the notable increase in FAC, she opted for timely induction again. She delivered a healthy, appropriate for gestational age (AGA), female infant weighing 3205 g (84th percentile) at 36 weeks and 5 days.

### Methods

The placentas of the subsequent pregnancies were histopathologically examined by a perinatal pathologist blinded to clinical information except for gestational age. For this report, after obtaining informed consent from the patient, we collected all available placental slides from different hospitals. Slides were reviewed by a perinatal pathologist (LEvdM) according to the Amsterdam Consensus Criteria.^4^

### Pathology findings

**FIGURE 1:**
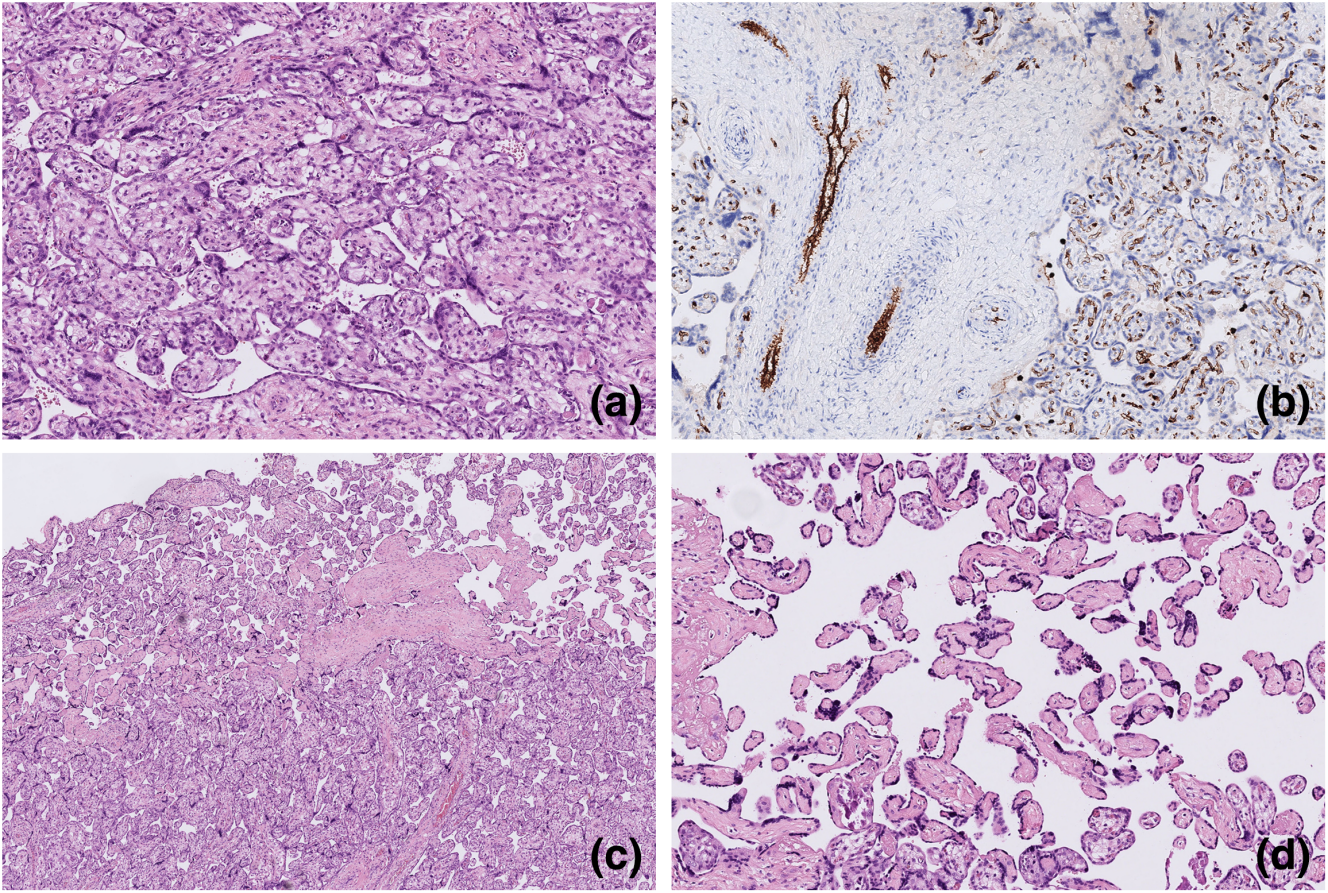
Pathology findings first placenta. (a) Delayed villous maturation (hematoxylin and eosin (H&E) stain), 10×. (b) Delayed villous maturation (CD15 stain: endothelium of fetal vessels is positive (brown staining)), 10×. (c) Avascular villi = fetal vascular malperfusion (H&E stain), 2×. (d) Fetal thrombosis (H&E stain), 10×.

Table S1 shows pregnancy and placental pathology characteristics. The first placenta had a relatively low weight (25th percentile, birthweight placenta weight ratio 9.2:1). The placenta showed strong signs of DVM as described by Jaiman et al.^9^ and Turowski et al.,^10^ fetal thrombosis, avascular villi, and infarcts with signs of (chronic) fetal hypoxia, Figure 1. There were no signs of MVM. There was a pattern of DVM with large immature villi and a continuous cytotrophoblast lining, centrally positioned fetal vessels, with CD15 positive immunostaining of the (fetal) endothelium. The number of terminal villi was insufficient; beyond 40% of the parenchyma that is usually present at term.^11^ Furthermore, the elevation of nucleated erythrocytes in the fetal circulation of this placenta indicated chronic fetal hypoxia resulting from this severe delayed maturation.^8^ In a placenta with a relatively light placental weight and less functional parenchyma because of delayed maturation, placental function is not adequate for a developing fetus at term. In the full‐term period, the combination of (1) DVM, (2) fetal thrombosis, and (3) multiple infarcts, is a frequently found cause of fetal demise.^12^

**FIGURE 2:**
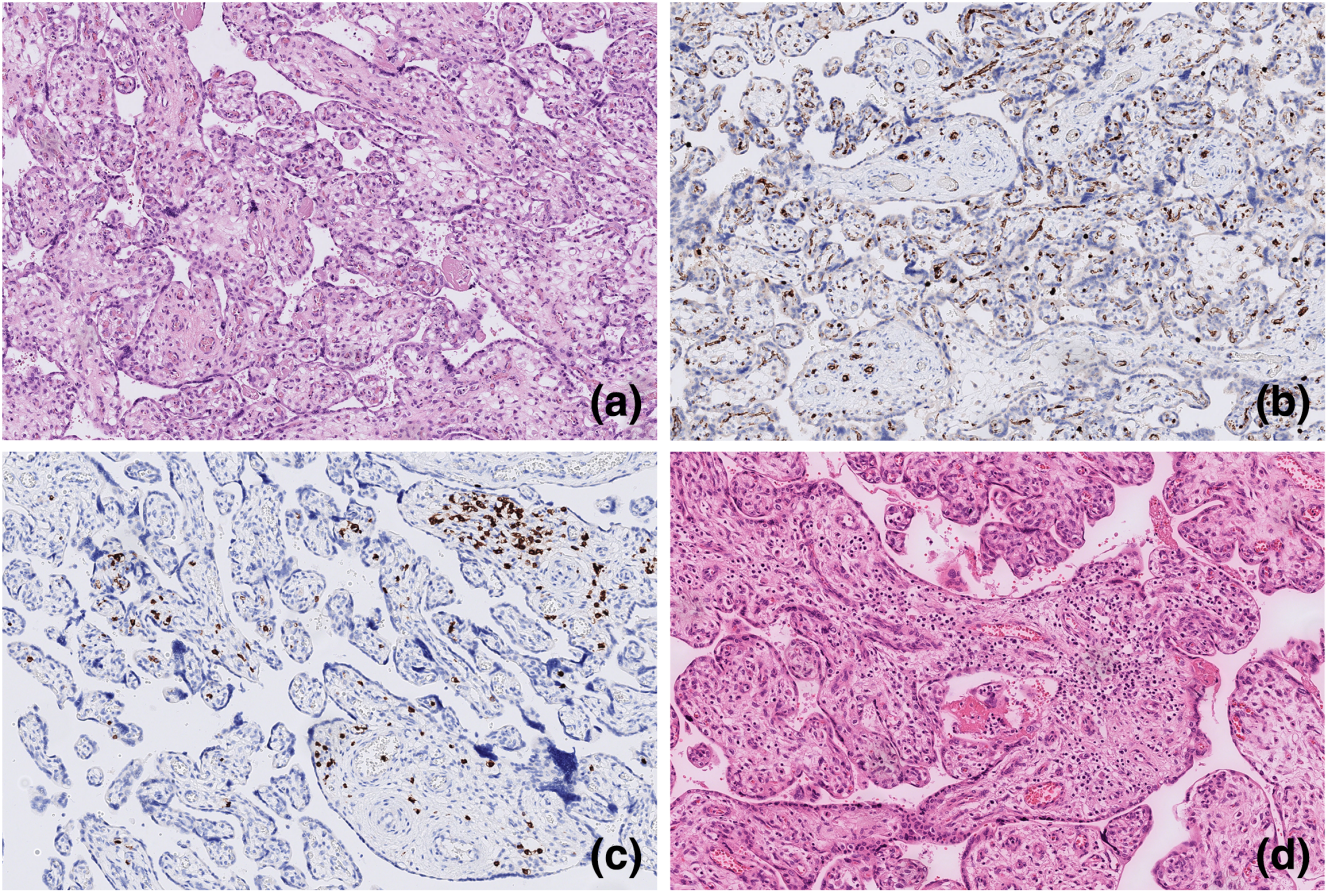
Pathology findings second placenta. (a) Delayed villous maturation (hematoxylin and eosin (H&E stain), 10×. (b) Delayed villous maturation (CD15 stain), 10×. (c) Chronic villitis of unknown etiology (CD3 stain), 10×. (d) Chronic villitis of unknown etiology (H&E stain), 10×.

The histological characteristics of the second placenta resembled those of the first. However, DVM was less pronounced. There were no signs of fetal thrombosis or infarcts. Remarkably, this placenta exhibited low‐grade chronic villitis of unknown etiology (VUE). Pathology findings are portrayed in Figure 2.

**FIGURE 3:**
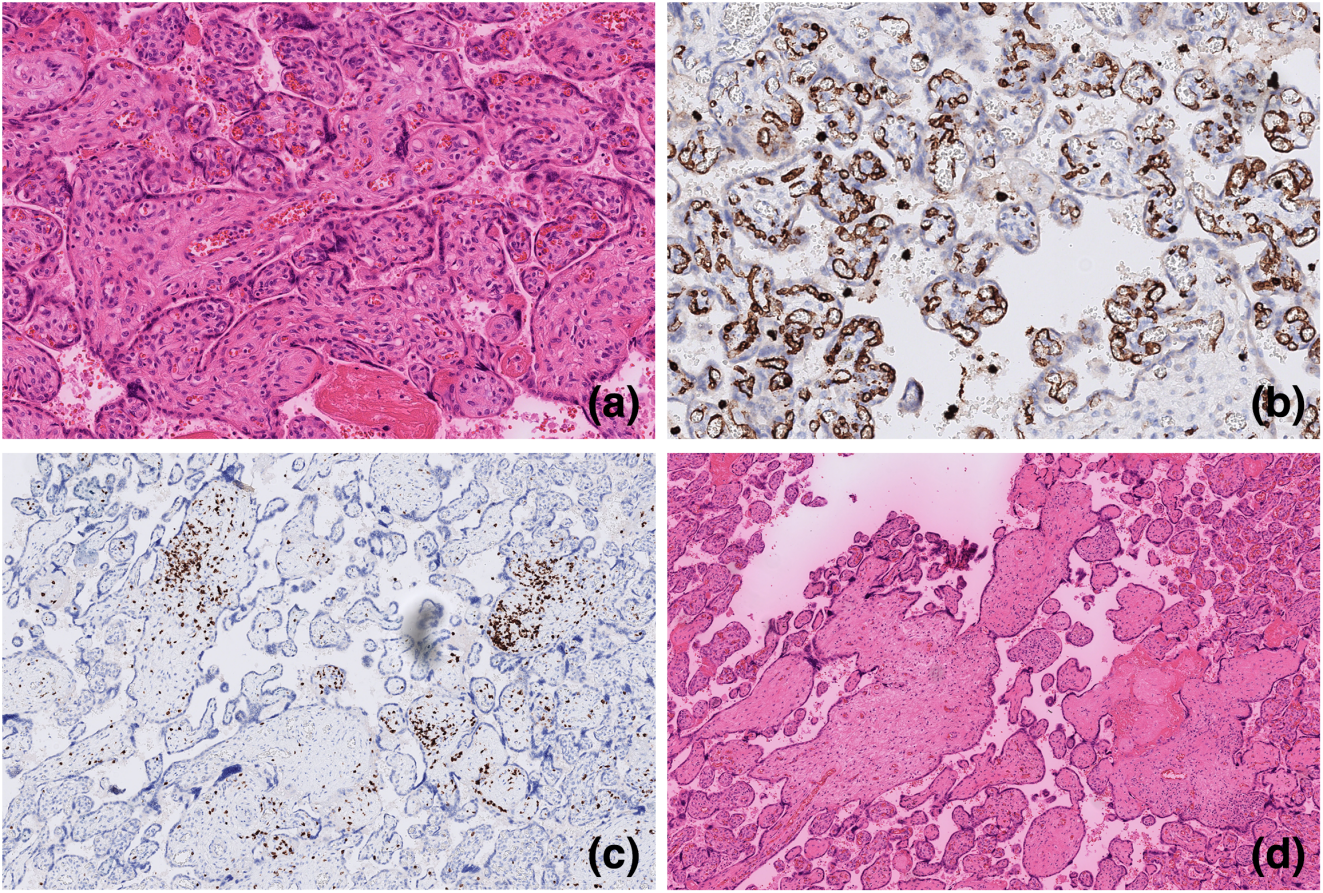
Pathology findings third placenta. (a) Delayed villous maturation (hematoxylin and eosin [H&E] stain), 10×. (b) Delayed villous maturation (CD15 stain), 10×. (c) Villitis of unknown etiology (CD3 stain), 5×. (d) Villitis of unknown etiology (H&E stain), 5×.

The third placenta showed a pattern comparable to the second placenta, with DVM and a more pronounced chronic VUE compared to the second placenta (Figure 3). Upon re‐review, unlike the second and third placentas, the first placenta did not exhibit any signs of VUE.

## DISCUSSION

Summarized, the pronounced signs of DVM, fetal thrombosis, and infarcts, along with signs of chronic fetal hypoxia in the relatively light first placenta, explained the fetal demise at 38 weeks and 4 days. By 38 weeks, this placenta could no longer meet the growing demands of this fetus.

### Delayed villous maturation

The placentas displayed significant positive CD15 and endothelial immunostaining, which is abnormal at term, indicating DVM.^9^, ^11^ DVM significantly contributes to sudden antenatal hypoxia (38%), unexplained fetal demise (9%), and adverse neonatal outcomes.^11^ The exact cause of DVM in these placentas remains unclear, and it was defined as idiopathic villous immaturity.

DVM mainly occurs in late pregnancy (>37 weeks), progressively impairing placental function and posing a significant threat if the fetus remains in utero after 37 weeks. Induction at 36 weeks arguably improved outcomes in the second and third pregnancies. The unexplained relatively low weight of the first placenta suggested limited reserve function, further compromised by parenchymal anomalies.

DVM is characterized by enlarged villi with deficient branching, dense collagenous fibers, reduced capillaries, and deficient vasculosyncytial membranes.^10^ DVM differs from distal villous hypoplasia, which lacks terminal villi, has an increased intervillous space, and is associated with MVM, low placental weight, and severe early onset FGR.^4^ DVM can only be diagnosed after birth, and conventional ultrasound techniques are not able to detect this parenchymal abnormality during pregnancy.^13^

For this case report, placental slides from all three pregnancies were reviewed by a perinatal pathologist who was blinded to pregnancy outcomes except for gestational age. The risk of bias was considered low because of the well‐established histological features of DVM, which were present in all three placentas (Figures 1, 2, 3).

### Differential diagnosis of DVM

DVM is associated with conditions such as GDM, metabolic disorders, obesity, chronic infection, nonimmunological hydrops fetalis, alpha‐thalassemia, and chronic fetal anemia.^10^ GDM was unlikely as a cause due to the absence of a clinical diagnosis; however, random glucose testing was not performed. Maternal metabolic disorders, obesity, and fetal conditions were ruled out as causes of DVM.

Early pregnancy viral infections (e.g., Rubella, Epstein Barr Virus, and Coxsackie B), auto‐immune disorders, and chronic villitis were also considered potential causes of DVM.^10^ Chronic villitis causes inflammation of the villi, which are essential for nutrient and oxygen exchange. When chronic villitis persists over time, it can disrupt placental function, leading to DVM. Although chronic VUE was found in the last two placentas, it was absent in the first. This could possibly be a sampling error since VUE recurrence risk is high.^14^ All births occurred in autumn and winter when seasonal influenza is common. Previous research suggests that there may be seasonal variation in VUE prevalence.^15^ Therefore, seasonal variation of VUE could not be ruled out.^14^, ^15^ She was vaccinated against coronavirus disease 2019 (COVID‐19) in her second and third pregnancy and had a confirmed COVID‐19 infection during the third and possibly influenza, though unconfirmed. However, the presence of low‐grade VUE in the second and third placentas suggested ongoing CI instead of AI.

### Macrosomia

Although there is a known association between DVM and fetal demise, primarily due to decreased placental reserve function, this association is typically seen in pregnancies with FGR and an impaired placental function due to placental insufficiency.^10^, ^13^ In this case report, the association of DVM and macrosomia is typical. In the first and second pregnancy, macrosomia was suspected. Based on pathology results and pregnancy characteristics, there was no indication of placental insufficiency, as seen in FGR. The fetus exhibited a growth pattern consistent with macrosomia, typical for DVM. Given the fetus's large size, DVM further impairing placental function may have contributed to a limited placental reserve function, ultimately leading to fetal demise. Because of increased nutritional and oxygen demands due to macrosomia, we hypothesize that the pregnancy ended in a fetal demise soon after DVM occurred. The exact pathophysiological mechanism of the occurrence of DVM in relation to macrosomia remains unknown.

### Birthweight placental weight ratio

Timely induction at 36 weeks in the second pregnancy resulted in a live LGA male infant with a birth weight >99th percentile and a relatively high birthweight placental weight ratio (7.4:1). This ratio, influenced by various factors, indicates the placenta's ability to support fetal growth. The relation between placental and fetal weight is not linear, since the exchange capacity of the placenta only partially depends on volume. At the extreme ends of the ratio, adverse perinatal outcomes are more commonly observed.^16^ A high ratio suggests a relatively large fetus compared to the placenta. The placental weight (p75‐90) of this LGA neonate suggests that other compensatory mechanisms might have come into play or timely delivery was beneficial.

Regardless of the presence of DVM and low‐grade VUE, the third pregnancy, after timely induction at 36 weeks, resulted in an AGA female infant (p84) with a birthweight to placental weight ratio of 7:1. The sex difference (female vs. male) might explain the (high) normal birth weight, potentially due to compensatory mechanisms.

### Low‐dose aspirin

A pragmatic approach of prophylactic low‐dose aspirin in the second and third pregnancies may have also improved placental function. Aspirin inhibits platelet aggregation and vasoconstriction, thereby improving placental function in pregnancies at high risk of placental insufficiency.^17^, ^18^, ^19^ While aspirin should be considered in pregnancies at high risk of placental insufficiency, primarily due to MVM or VUE, its effect on placental function in the case of DVM remains unproven.^19^, ^20^ Nevertheless, empirical prophylactic treatment with aspirin for DVM could be considered.^20^ Future research should focus on stratifying participants based on placental lesions to evaluate the effectiveness of interventions such as low‐dose aspirin prophylaxis.^21^

### Induction of labor

We hypothesize that timely induction arguably may have contributed to better outcomes in the second and third pregnancies, as the limited placental reserve caused by DVM and villitis could increase the risk of fetal demise at later gestational ages. However, the exact impact of induction of labor remains unclear. Expert reviews by Redline and Matsika suggest different management strategies for subsequent pregnancies following DVM, including screening for diabetes, recommending weight loss, performing third‐trimester fetal movement counts, but also considering delivery before 40 weeks, though specific timing is not further defined.^19^, ^20^ Additionally, both reviews suggest elective early delivery for subsequent pregnancies following VUE.^19^, ^20^ In case of DVM and villitis, placental function decreases over time. In the first trimester, the placenta is relatively big compared to the small fetus. In the second trimester, the balance is still in favor of the fetus, but this gradually changes toward the third trimester. During the third trimester, the placenta is relatively small and further maturation of villi is necessary to increase functional parenchyma to support the increasing fetal demands. At 38 weeks, when the fetal demise occurred, the suboptimal placenta could no longer meet the fetus's demands. Timely induction may have prevented this in the subsequent pregnancies. While early delivery may help prevent hypoxia, its benefits could be outweighed by the risks associated with (relative) prematurity.^22^ The decision for indicated (preterm) birth must balance the low likelihood but significant impact of stillbirth against the high risk and relatively lower impact of the fetus's unmet needs for further maturation.^22^ This should be an individualized decision, considering the clinical context and underlying reason for DVM, and should be made carefully in consultation with the patient, obstetricians, and neonatologists.

### Recurrence risk

DVM has a risk of recurrence of around 5%, while VUE recurrence varies between 30% and 70% depending on severity.^10^, ^14^ Since no definitive histological or clinical features can reliably predict the recurrence of these placental lesions, and the exact consequences of these lesions are difficult to predict, adequate fetal monitoring is essential in subsequent pregnancies.

## CONCLUSION

The availability of three placentas from a single patient is rare and valuable. This study highlights the importance of structured placental examination by a perinatal pathologist. Referral for placental review in a specialized center should be encouraged when expertise is lacking. A reliable pathology conclusion is essential to improve monitoring and management in future pregnancies. Arguably, our interventions in the second and third pregnancies, including low‐dose aspirin and timely induction, resulted in successful outcomes, despite similar placental lesions and suboptimal placental function due to these lesions. However, induction of labor should always be approached cautiously and individually balanced, as (relative) prematurity may increase the risk of perinatal complications postpartum. A better understanding of the pathophysiology of DVM could improve management strategies in subsequent pregnancies. This case report underscores the importance of histologic placental examination following complicated pregnancies.

## AUTHOR CONTRIBUTIONS

**M. C. Marijnen:** Conceptualization; investigation; methodology; writing – original draft. **W. Ganzevoort:** Conceptualization; supervision; writing – review and editing. **S. J. Gordijn:** Conceptualization; supervision; writing – review and editing. **L. E. van der Meeren:** Conceptualization; investigation; methodology; supervision; visualization; writing – review and editing.

## CONFLICT OF INTEREST STATEMENT

The authors declare no conflict of interests for this article.

## ETHICS STATEMENT

Ethics approval was not required. Written informed consent was obtained from the patient.

## Supporting information


**Table S1.** Pregnancy and placental characteristics.

## Data Availability

Data sharing not applicable to this article as no datasets were generated or analyzed during the current study.
