# Supplementary material for: Delayed villous maturation with and without fetal demise: A case report of three successive pregnancies
Source: J Obstet Gynaecol Res. 2025 May 6;51(5):e16308. doi: 10.1111/jog.16308 (PMC12056347; doi:10.1111/jog.16308)
Supplement: Supplementary file 1 — Table S1. Pregnancy and placental characteristics. [file JOG-51-0-s001.docx]

APPENDIX

**Table S1**. *Pregnancy and placental characteristics*

| *Pregnancy* | First pregnancy | Second pregnancy | Third pregnancy |
| --- | --- | --- | --- |
| *Pregnancy characteristics* |  |  |  |
| *Gestational age (weeks + days)* | 38 + 4 | 36 + 2 | 36 + 5 |
| *Live birth* | No | Yes | Yes |
| *Sex* | Male | Male | Female |
| *Birthweight (grams)* | 4,065 | 3,960 | 3,205 |
| *Birthweight percentile* | p95 | >p99 | p84 |
| *Placenta characteristics* |  |  |  |
| *Placental weight (grams)* | 433 | 532 | 455 |
| *Placental weight percentile* | p25 | p75-90 | p25-50 |
| *Birthweight placental weight ratio :1* | 9.2 | 7. 4 | 7 |
| *Histology* | Delayed villous maturation;  Fetal vascular malperfusion | Delayed villous maturation;  Chronic villitis | Delayed villous maturation;  Chronic villitis |
